# Tunnel Structure Enhanced Polysulfide Conversion for Inhibiting “Shuttle Effect” in Lithium-Sulfur Battery

**DOI:** 10.3390/nano12162752

**Published:** 2022-08-11

**Authors:** Xiaotong Guo, Xu Bi, Junfeng Zhao, Xinxiang Yu, Han Dai

**Affiliations:** 1 Laboratory of Advanced Light Alloy Materials and Devices, Yantai Nanshan University, Longkou 265713, China; 2 Yulong Petrochemical Co., Ltd., Longkou 265700, China

**Keywords:** lithium sulfur battery, manganese oxides, tunnel structure, cathode materials, shuttle effect

## Abstract

The Lithium sulfur (Li-S) battery has a great potential to replace lithium-ion batteries due to its high-energy density. However, the “shuttle effect” of polysulfide intermediates (Li_2_S_8_, Li_2_S_6_, Li_2_S_4_, etc.) from the cathode can lead to rapid capacity decay and low coulombic efficiency, thus limiting its further development. Anchoring polysulfide and inhibiting polysulfide migration in electrolytes is one of the focuses in Li-S battery. It is well known that polar metal oxides-manganese oxides (MnO_2_) are normally used as an effective inhibitor for its polysulfide inhibiting properties. Considering the natural 1D tunnel structure, MnO_2_ with three kinds of typical tunnel-type were screened to study the effects of the tunnel size on the adsorption capacity of polysulfide. We found that MnO_2_ with larger tunnel sizes has stronger chemisorption capacity of polysulfide. It promotes the conversion of polysulfide, and corresponding cathode exhibits better cycle reliability and rate performance in the cell comparison tests. This work should point out a new strategy for the cathode design of advanced Li-S battery by controlling the tunnel size.

## 1. Introduction

With increasing demand of chemical energy storage, the lithium sulfur (Li-S) battery with a high theoretical capacity of 1675 mAh g^−1^, is considered to be a promising candidate to replace the state-of-the-art lithium-ion batteries [1,2,3]. Meanwhile, sulfur is abundant, environmentally friendly and cost effective, which fully meets the requirements of secondary energy storage power [4,5,6]. However, the practical application of the Li-S battery is still hindered by the rapid capacity decay, low coulombic efficiency and poor rate performance, which are mostly due to the “shuttle effect” [7,8,9,10,11]. Recently, meso/microporous carbon [12,13,14], carbon nanotubes [15,16,17] and other porous carbon-based materials which have good conductivity and chemical stability, have been applied to suppress the shuttle of polysulfide, keep good conductivity and improve sulfur utilization. However, their ability for polysulfide adsorption is very limited due to their nonpolar nature.

As for enhancing the polysulfide adsorption ability and suppressing the “shuttle effect”, metal oxides are noticed because of their strong polar–polar chemical interaction with polysulfide [18,19,20,21,22,23,24,25]. More recently, lots of literature has reported that anchoring polysulfide by chemical reaction is much more promising and metal oxides such as SiO_2_, V_2_O_5_, Al_2_O_3_, TiO_2_, and MnO_2_ could significantly improve cycling performance and realize high-loading sulfur in Li-S battery [26,27]. Among known metal oxides which are used as cathode materials, MnO_2_ has the most potential in the Li-S battery due to its abundant resources, low cost, and nontoxicity. The previous research indicates MnO_2_ has a particularly strong adsorption capacity for polysulfide compared to carbon materials and other metal oxides [28]. Moreover, MnO_2_ has a large variety of crystal structures including α, β, δ, λ and so on, depending on the edge-sharing and angle-sharing MnO_6_ octahedron. Thus, 1D tunnel, 2D layer, and 3D mesh structure can be formed [29,30,31,32,33]. δ-MnO_2_ as a layered structure can be used as an effective sulfur host material. Nazar’s group firstly reported that δ-MnO_2_ can be considered as a remarkable chemical inhibitor for polysulfide based on mediating polysulfides redox [34]. For the tunnel-type MnO_2_, Ni and coworkers prepared γ-MnO_2_ covered with sulfur nanospheres, forming a core–shell structure to trap polysulfide through physical and chemical effects [35]. Zhang and researchers fabricated Mo-doping γ-MnO_2_ to accelerate the transformation of polysulfide [36]. Wang and coworkers have synthesized highly ordered mesoporous β-MnO_2_ to encapsulate sulfur and found that the thin mesoporous walls could provide short diffusion distances for Li ions [37]. These hybrid structures can physically and chemically encapsulate polysulfide and enhance the electrochemical performance of sulfur cathode. The studies above have reported synthesizing tunnel-type MnO_2_ by in-situ recombination or ion doping and its application as sulfur host, but little work has focused on the effects of tunnel sizes on the polysulfide conversion abilities.

Herein, MnO_2_ with three different tunnel sizes have been screened and prepared by hydrothermal reaction to investigate the effects of tunnel sizes on the polysulfide conversion. It is found that MnO_2_ with a larger tunnel size has stronger chemisorption capacity of polysulfide, and therefore is more favorable to inhibiting the polysulfide shuttle. In addition, the larger tunnel size possesses faster reaction kinetics in the redox reaction in Li-S battery, hence larger-tunnel-size-MnO_2_ based cathode exhibits better cycle reliability and rate performance in the cell tests. This work should provide a new perspective for the cathode design of advanced Li-S battery.

## 2. Materials and Methods

### 2.1. Synthesis of Manganese Oxides

**Figure 1:**
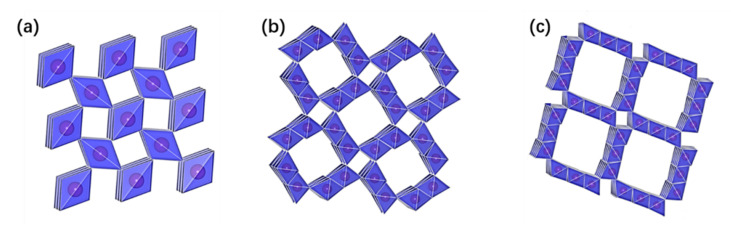
Tunnel structure of MnO_2_: (**a**) β-MnO_2_ (1 × 1, 2.3 Å × 2.3 Å), (**b**) α-MnO_2_ (2 × 2, 4.6 Å × 4.6 Å), (**c**) t-MnO_2_ (3 × 3, 6.9 Å × 6.9 Å).

Figure 1 illustrates the theoretical structures of the three tunnel-types of MnO_2_ and how each tunnel phase is conventionally named as M × N, where M and N stand for the number of the MnO_6_ octahedra constituting the height and width of the tunnel, respectively [38,39]. Depending on the number of MnO_6_ units in the MnO_2_, the tunnel size increases from β-MnO_2_ (1 × 1 tunnel) to α-MnO_2_ (2 × 2 tunnel), and to todorokite MnO_2_ (3 × 3 tunnel) [40,41,42,43].

The β-MnO_2_ was obtained as follows: First, 1.0 g of MnSO_4_·H_2_O was dissolved in 60 mL of distilled deionized water, and then 0.37 g KMnO_4_ was added into the solution with stirring. Then, the slurry was transferred into a Teflon-lined stainless-steel autoclave and heated at 120 °C for 12 h. The resulting product was washed and dried at 60 °C overnight.

The α-MnO_2_ was prepared as follows: Firstly, 3.67 g of MnAC_2_·4H_2_O and 2.5 mL of CH_3_COOH were dissolved in 35 mL deionized water with stirring. Then, 40 mL of 2.17 g KMnO_4_ was added to the above mixture. Finally, the mixed solution was maintained at 100 °C for 24 h in a Teflon-lined autoclave. The resulting product was collected by centrifugation, washed with deionized water and dried at 100 °C overnight.

The todorokite MnO_2_ (t-MnO_2_) was synthesized as follows: First, 30 mL 6.0 M of NaOH aqueous solution was dropped into the solution (20 mL) containing 1.7 g of MnSO_4_·H_2_O with stirring. Then, 0.35 g of MgSO_4_·7H_2_O and 1.90 g of K_2_S_2_O_8_ were added to the above mixture. After stirring for 3 h at room temperature and being washed with distilled water, the wet sample was dispersed in 300 mL of 1.0 M of MgCl_2_·6H_2_O and stirred for 24 h for ion exchange. The obtained mixture was transferred into a Teflon-lined autoclave and heated at 160 °C for 24 h. The resulting mixture was filtered and washed with distilled water. Lastly, the product was dried at 100 °C for 12 h.

### 2.2. Preparation of the Sulfur Composites

Sulfur and manganese oxides were mixed with a ratio of 70:30. The mixture was then transferred into a sealed stainless-steel vial and heated at 155 °C for 12 h in an oven.

### 2.3. Cell Assembling and Testing

The cathodes were prepared with 60 wt% active material, 30 wt% Super P carbon, and 10 wt% polyvinylidene fluoride (PVDF) binder and the slurry was casted onto Al foil current collector. The electrodes were dried at 60 °C in a vacuum for 24 h. The CR2032 coin cells with lithium metal (counter electrode) were fabricated. Sulfur cathode size is d = 12 mm, pure sulfur loading is ~1 mg cm^−2^, and overall sulfur content is 42 wt%. The electrolyte was 1.0 M LiTFSI in dioxolane/dimethoxyethane solvent (DOL/DME volume ratio1:1) with 2 wt% lithium nitrate (LiNO_3_) as an additive and PE was used as the separator. Galvanostatic measurements were carried out between 1.8 and 2.8 V (vs. Li/Li^+^) on a Land CT2001A system (LANHE, Wuhan, China). The cyclic voltammetry (CV) experiments were performed with a CHI600E electrochemical workstation (CH, Shanghai, China) at a scanning rate of 0.1 mV s^−1^ between 1.7 and 2.8 V.

### 2.4. Polysulfide Adsorption Test

Typically, 5 mM Li_2_S_6_ solution was prepared by the reaction Li_2_S with S in DOL and DME (*v/v* = 1:1). Then three samples of the same weight (5 mg) were added into three glass vials, respectively, and dispersed in 2 mL Li_2_S_6_ solution. Optical images were taken to compare the adsorption ability.

### 2.5. Structure Characterization

The morphology was examined by using scanning electron microscopy (SEM, JSM-2100F, JEOL, Tokyo, Japan). XRD patterns were collected using a D/max-TTR III (Rigaku Corporation, Shibuya-ku, Japan) with Cu Ka radiation, 40 kV, 200 mA). Nitrogen adsorption/desorption isotherms were performed on a Quantachrome Autosorb-IQ system (Quantachrome Instruments, Boynton Beach, FL, USA). The nanostructures of manganese oxides were characterized by high-resolution transmission electron microscopy (HRTEM, JEOL, Tokyo, Japan, 2010). For XPS, the samples were sealed in a vial before being quickly transferred to the chamber of an ultra-high vacuum Imaging XPS Microprobe system for analysis (Thermo Scientific ESCALAB 250Xi, Waltham, MA, USA).

## 3. Results

**Figure 2:**
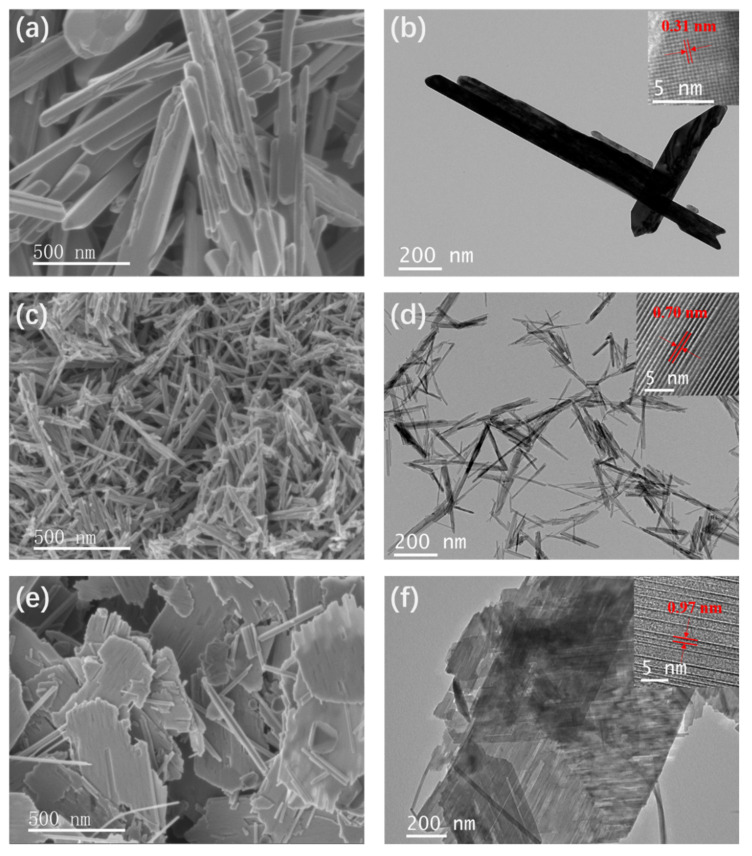
Morphological characterization: (**a**,**c**,**e**) SEM images of β-MnO_2_, α-MnO_2_ and t-MnO_2_, respectively; (**b**,**d**,**f**) TEM images of β-MnO_2_, α-MnO_2_ and t-MnO_2_, respectively (Insert: HRTEM images).

As illustrated in Figure 2, the morphology of prepared three tunnel-type MnO_2_ was carefully characterized. In the Figure 2a,c, the SEM images clearly showed the nanofiber morphology of the β-MnO_2_ and α-MnO_2_, with a length ranging from tens of nanometers to hundreds of nanometers. No extra phase was found on the surface of nanofiber. Because of the different reaction mechanism, the synthesized t-MnO_2_ is nano-flake and with some long nanoribbons (Figure 2e). HTEM was used to measure the tunnel sizes of β-MnO_2_, α-MnO_2_ and t-MnO_2_. In the Figure 2b, the HRTEM image shows the interlayer distance is 0.31 nm, which agrees with the (110) plane in the crystal structure of β-MnO_2_, and hence the existence of 1 × 1 tunnel is confirmed. The clear lattice fringes in Figure 2d show that the crystal plane spacing of α-MnO_2_ is 0.70 nm, which corresponds to the (110) plane. For the t-MnO_2_ structure, the crystallinity can be observed from the distinct lattice fringes in HRTEM image (Figure 2f). The interlayer distance of 0.97 nm corresponds to the (100) plane at an angle of 9.2°, thereby confirming the existence of 3 × 3 tunnel.

**Figure 3:**
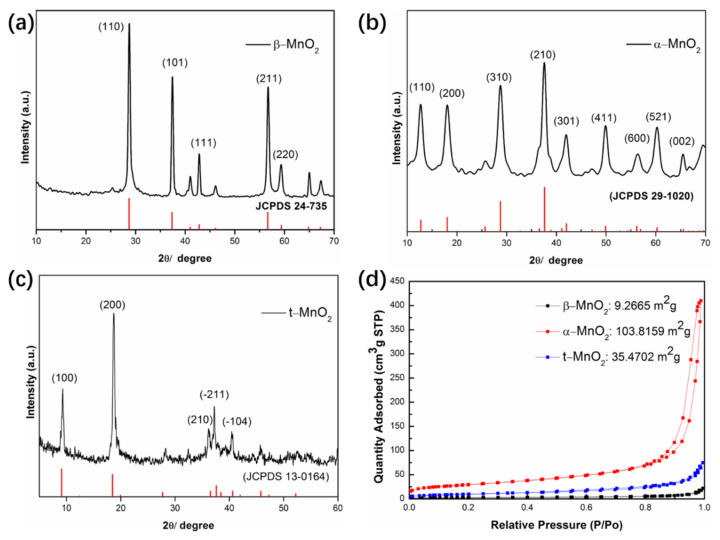
Material characterization: (**a**) XRD pattern of the β-MnO_2_; (**b**) XRD pattern of α-MnO_2_; (**c**) XRD pattern of t-MnO_2_; (**d**) Nitrogen adsorption-desorption isotherm of the synthesized MnO_2_.

X-ray diffraction (XRD) measurement was used to confirm the crystal structure of synthetic tunnel-type MnO_2_ (Figure 3). As shown in Figure 3a, the diffraction pattern for the β-MnO_2_ has five sharp peaks at 28.7°, 37.3°, 42.8°, 56.6° and 59.3°, corresponding to (110), (101), (111), (211), and (220) of pyrolusite (JCPDS: 24-735), respectively. For the synthesized α-MnO_2_, peaks appearing in the Figure 3b can be well indexed to the pure tetragonal cryptomelane structures of α-MnO_2_ (JCPDS card 29-1020). In addition, as for the XRD pattern of t-MnO_2_ (Figure 3c), peaks at 9.1°, 18.5°, 36.5°, 37.6° and 38.4°, also well coincide with the standard crystallographic tables JCPDS card 13-0164, showing the todorokite-type with the monoclinic phase. Thus, the MnO_2_ of three different tunnel sizes were successfully synthesized through simple hydrothermal reaction without impurities. Figure 3d presents the N_2_ adsorption/desorption analysis of the MnO_2_ samples. The BET (Brunauer-Emmett-Teller) surface area and total pore volume of β-MnO_2_, α-MnO_2_ and t-MnO_2_ presented in Figure S1 are 9.27 m^2^/g and 0.018 cm^3^/g, 103.82 m^2^/g and 0.38 cm^3^/g, 35.47 m^2^/g and 0.074 cm^3^/g, respectively. As a result, few micropores are formed on these three types of MnO_2_. The reason why α-MnO_2_ has the largest BET surface area should be attributed to the smaller nanofiber morphology.

**Figure 4:**
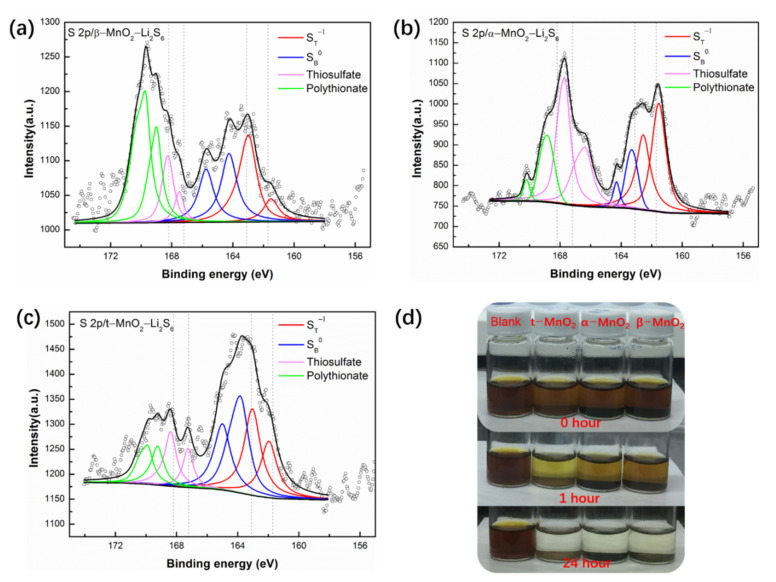
(**a**) S2p XPS spectra of β-MnO_2_-Li_2_S_6_; (**b**) S2p XPS spectra of α-MnO_2_-Li_2_S_6_; (**c**) S2p XPS spectra of t-MnO_2_-Li_2_S_6_; (**d**) Polysulfides adsorption test.

To compare the polysulfide adsorption capacity of three tunnel-type MnO_2_, polysulfide adsorption test was performed. Typically, three samples of the same weight (5 mg) were added into three glass vials, respectively, and dispersed in 2 mL Li_2_S_6_ solution. After 1 h adsorption, the solution changed from dark-yellow to light yellow (Figure 4d). Obviously, the solution containing t-MnO_2_ was noticeably lighter in color than other solutions, which indicates t-MnO_2_ has greater ability to adsorb polysulfide faster. It is usually believed that larger surface area means stronger adsorption capacity, but the BET surface area of t-MnO_2_ is 35.47 m^2^g, smaller than half of that of α-MnO_2_ (103.82 m^2^g). As a result, adsorption capacity of these atomic tunnels of MnO_2_ were not well reflected by normal surface area tests. Similar adsorption independent of specific surface area by MnO_2_ on methylene blue has also been reported by ref. [44], which apparently exhibits the special adsorption properties. Therefore, the stronger adsorption of t-MnO_2_ of polysulfide mentioned above should be partially attributed to the special adsorption properties of the larger tunnel size of the polysulfide.

X-ray photoelectron spectroscopy (XPS) analysis was used to find out the absorption type between polysulfide and tunnel-type MnO_2_. By examining the specimens taken out from the Li_2_S_6_ solution after the adsorption test, it is found that all the S2p XPS spectra of β-MnO_2_, α-MnO_2_ and t-MnO_2_ with polysulfides reveal four types of sulfur environment, as shown in Figure 4a–c. In the lower-binding-energy region from 163 eV to 165 eV, two pairs of S2p peaks refer to the same terminal (ST) and bridging sulfur (SB) peak, coming from the sulfides and polysulfides. The peaks in the higher-binding-energy region between 171 and 166 eV correspond with the binding energy of thiosulfate and polythionate species, which arise from the redox reaction between Li_2_S_6_ and MnO_2_ [34]. Therefore, the chemisorption process occurs in such a way: the tunnel-type MnO_2_ reacts with polysulfide and converts polysulfide to thiosulfate and polythionate, thereby anchoring polysulfide on the surface of MnO_2_.

**Figure 5:**
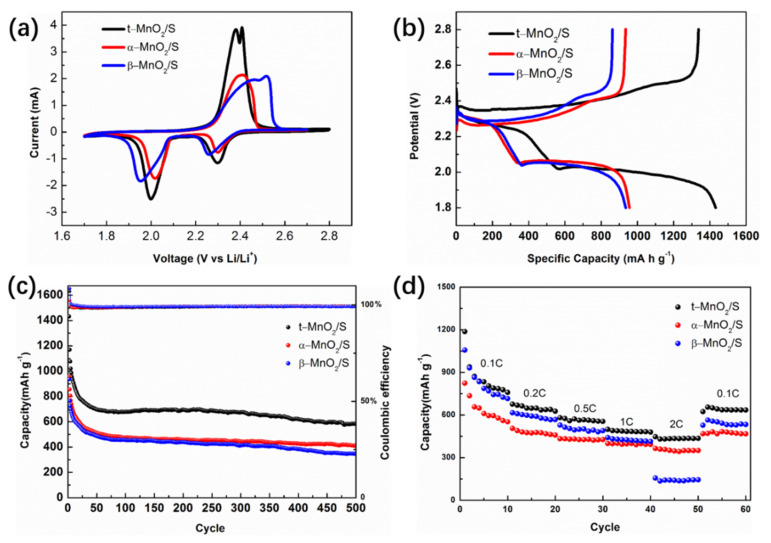
Electrochemical performance of Li-S battery: (**a**) CV profiles; (**b**) the first charge-discharge curves; (**c**) long cycle performance; and (**d**) rate performance.

Considering the excellent inhibiting effects on the “shuttle effect” by the tunnels of MnO_2_, then, electrochemical experiments with the cathodes of tunnel-type MnO_2_ were performed. Cyclic voltammograms (CV) of cells in the first cycle were shown in Figure 5a. Two typical cathodic peaks of the three cathodes were all observed at ~2.3 V and ~2.0 V, which could be ascribed to the reduction process of sulfur. These two peaks are, respectively, assigned to the transformation of S_8_ to the long-chain polysulfide and then the reduction of long-chain sulfide species to solid products (Li_2_S_2_/Li_2_S). When sweeping back, two sharp peaks of t-MnO_2_/S cathode were found at 2.35 V and 2.4 V, which is mainly due to the oxidation of short-chain sulfide to polysulfide and S_8_. Similarly, the β-MnO_2_/S cathode exhibits two peaks located at 2.44 V and 2.5 V. However, for the α-MnO_2_/S cathode, only one peak was observed at 2.4 V, indicating the complete conversion of Li_2_S and polysulfides into element sulfur. A large number of studies reported that it was a common phenomenon in the first CV scan of Li-S battery [45,46]. Notably, the peak current of t-MnO_2_/S electrode is greater than that of the α-MnO_2_/S and β-MnO_2_/S electrodes, which represents higher kinetics and reversibility of redox reaction. It makes sense that ions diffuse more easily in larger channels or more open structures. Moreover, the larger the cavity of tunnel-type MnO_2_, the higher the ionic conductivity [47]. Relevant studies on kinetics of MnO_2_ have already been widely reported [37,48]. Thus, the t-MnO_2_ with 3 × 3 tunnel could provide wider pathways and more spacious cavities for the incorporation of Li^+^ ions into the material bulk than other smaller tunnel sizes.

Figure 5b shows the typical galvanostatic discharge/charge profiles of the Li-S battery at 1 C rate. For the initial cycle, the t-MnO_2_/S based cell delivers an excellent discharge capacity of 1431.9 mAh g^−1^ at 1 C, which is significantly larger than the 935.9 mAh g^−1^ of β-MnO_2_/S and 956.7 mAh g^−1^ of α-MnO_2_/S cathodes. The maximum discharge capacity reflects the utilization degree of sulfur when the battery carries on galvanostatic discharge. In order to enhance sulfur utilization, the sulfur in the electrode needs to be fully exposed to lithium ions and electrons, so as to effectively reduce to the lithium polysulfide species. The high initial discharge of t-MnO_2_/S cathode is due to the larger tunnel structure, which enhances sulfur utilization. In addition, the first discharge curves exhibited two plateaus at ~2.0 V and ~2.3 V, which coincides with the CV results. Upon charge process, the platform voltage is also consistent with the corresponding CV results.

In order to directly demonstrate the effects of strong chemisorption on polysulfide by tunnel-type MnO_2_, we further studied the cycle performance and rate capacity of Li-S cells assembled with β-MnO_2_/S, α-MnO_2_/S and t-MnO_2_/S as cathodes. As shown in the Figure 5c, the long-term cycle stability of Li-S battery in terms of discharge capacity and coulombic efficiency was also studied at 1 C. The cycle performance of the three electrodes was compared, and the cell with t-MnO_2_/S was found to have the best cycle stability in the long cycle. The residual capacity maintains in 583.7 mAh g^−1^ even over 500 cycles, which is still superior to 408 mAh g^−1^ of α-MnO_2_/S and 345 mAh g^−1^ of β-MnO_2_/S. Moreover, impressively, the coulombic efficiency of the t-MnO_2_/S based battery remain over 99% during the long-term cycling, which indicates its excellent intrinsic reversibility. In addition, SEM images of the discharged MnO_2_/S cathode after 100 cycles under 1 C, which further proves the stable structure of t-MnO_2_/S cathode to buffer the volume changes during repeated lithiation processes. Therefore, the enhanced chemisorption of t-MnO_2_ to polysulfide effectively promotes anchoring polysulfide and inhibiting the shuttle of polysulfide, and thus greatly improving the cyclic stability and coulomb efficiency of the Li-S battery.

The rate performance of the cell was obtained in Figure 5d. It is noteworthy that the cell with t-MnO_2_/S shows a superior rate capability of 447 mAh g^−1^ at 2 C, which is 82 mAh g^−1^ higher than the specific discharge capacity of α-MnO_2_/S cathodes and 291 mAh g^−1^ higher than that of β-MnO_2_/S cathodes. In addition, t-MnO_2_/S cathode shows 1371, 676, 581, 498 mAh g^−1^ at 0.1, 0.2, 0.5 and 1 C, respectively. The preferable rate capability of t-MnO_2_/S based cell should mainly be attributed to the larger 3 × 3 tunnel structure, which accelerates the conversion of polysulfide and improves the reaction kinetics at higher current density.

## 4. Conclusions

In summary, MnO_2_ with three different types of tunnel sizes were designed to investigate their chemisorption capacity for polysulfide. MnO_2_ with larger tunnel size shows stronger chemisorption capacity through the redox reaction. The larger tunnel size of t-MnO_2_ not only anchors and accelerates the conversion of polysulfide but also possesses faster reaction kinetics in Li-S battery. Thus, the t-MnO_2_ based battery, with an ultrahigh initial capacity of 1431.9 mAh g^−1^ and a capacity of 583.7 mAh g^−1^ after 500 cycles, shows better cell performance and higher coulombic efficiency than the other two electrodes. Apparently, this work will bring some new concepts and strategies to the material selection and structure design for advanced Li-S battery.

## Data Availability

Data available in a publicly accessible repository.

## Supplementary Materials

The following supporting information can be downloaded at: https://www.mdpi.com/article/10.3390/nano12162752/s1. Figure S1: Pore–size distribution obtained using the Barrett–Joyner–Halenda (BJH) method; Figure S2: EX situ SEM images of the discharged MnO_2_/S cathodes after 100 cycles under 1 C. (a,b) t-MnO_2_/S cathode; (c,d) α-MnO_2_/S cathode; and (e,f) β-MnO_2_/S cathode. Reference [49] is cited in Supplementary Materials.
