# Supplementary material for: Tunnel Structure Enhanced Polysulfide Conversion for Inhibiting “Shuttle Effect” in Lithium-Sulfur Battery
Source: Nanomaterials (Basel). 2022 Aug 11;12(16):2752. doi: 10.3390/nano12162752 (PMC9415869; doi:10.3390/nano12162752)
Supplement: Supplementary file 1 [file nanomaterials-12-02752-s001.zip › nanomaterials-1858663-supplementary.pdf]

nanomaterials

Supplementary Materials

Tunnel Structure Enhanced Polysulfide Conversion for Inhibiting "Shuttle Effect" in Lithium-Sulfur Battery

Xiaotong Guo [1,2], Xu Bi [1,2], Junfeng Zhao [1], Xinxiang Yu [1] and Han Dai [1,*]

[1] Laboratory of Advanced Light Alloy Materials and Devices, Yantai Nanshan University, Longkou 265713, China
[2] Yulong Petrochemical Co., Ltd., Longkou 265700, China
* Correspondence: daihan1985@189.cn

The total pore volume of β-MnO_2, α-MnO_2 and t-MnO_2 is 0.018 cm^3/g, 0.38 cm^3/g and 0.074 cm^3/g, respectively. According to the test results, few micropores are formed on these three types of MnO_2, which indicates that the specific surface area values measured by N_2-sorption isotherms of the MnO_2 samples are relatively accurate.

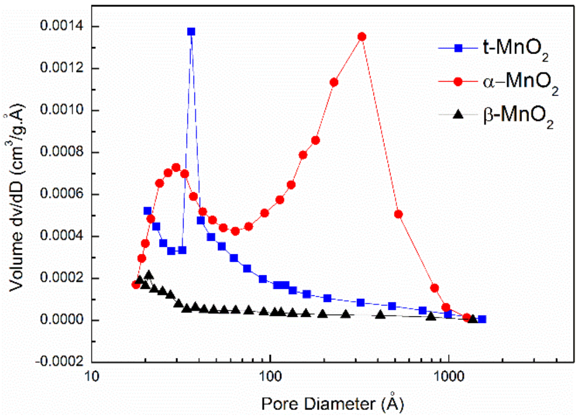

Figure S1. Pore–size distribution obtained using the Barrett–Joyner–Halenda (BJH) method.

The morphological analyses of cycled cathodes were analyzed by the SEM (Fig.S2). Figure S2 shows the SEM images of the discharged MnO_2/S cathode after 100 cycles under 1 C. It can be found that the t-MnO_2/S cathode has good surface integrity, the crack on the surface is smaller and shallower compared with β-MnO_2/S and α-MnO_2/S cathodes, indicating the structure of t-MnO_2/S cathode is more stable to buffer the volume changes during repeated lithiation processes. Indeed, the particles on the surface of cycled t-MnO_2/S cathode like being coated with smooth and uniform SEI film (Figure S1.b), which is crucial for improved cycle performance of Li-S cell [1]. There is a little regret here, XPS failed to get valid data. However, it can be expected that the t-MnO_2/S could be more stable to maintain the original material state due its better cell performance and more stable surface state of cycled cathode.

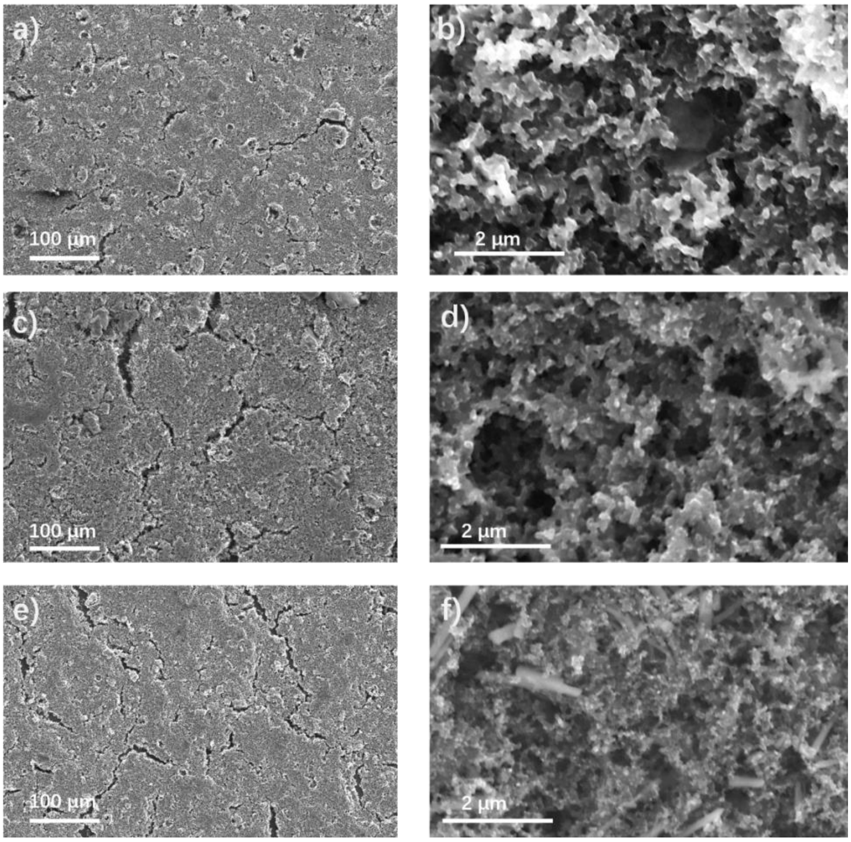

Figure S2. EX situ SEM images of the discharged MnO_2/S cathodes after 100 cycles under 1 C. (**a**,**b**) t-MnO_2/S cathode; (**c**,**d**) α-MnO_2/S cathode; and (**e**,**f**) β-MnO_2/S cathode.

References

1. He, B.; Rao, Z.; Cheng, Z.; Liu, D.; He, D.; Chen, J.; Miao, Z.; Yuan, L.; Li, Z.; Huang, Y. Rationally Design a Sulfur Cathode with Solid-Phase Conversion Mechanism for High Cycle-Stable Li–S Batteries. *Advanced Energy Materials* **2021**, *11*, 2003690, doi:https://doi.org/10.1002/aenm.202003690.
